# The “D&I Bridge”: introducing a teaching tool to define the D, the I, and the why

**DOI:** 10.1186/s43058-024-00558-z

**Published:** 2024-02-27

**Authors:** Sara J. Becker, Kira DiClemente-Bosco, Kelli Scott, Sarah A. Helseth, Zabin Patel-Syed, Dennis H. Li

**Affiliations:** Center for Dissemination and Implementation Science, Northwestern University Feinberg School of Medicine, 633 N St Clair Street, Chicago, IL 60611 USA

**Keywords:** Dissemination science, Implementation science, Teaching, Public health

## Abstract

Interest in learning dissemination and implementation (D&I) science is at an all-time high. As founding faculty and fellows of a new center focused on D&I science, we have found that non-specialist researchers and newcomers to D&I science often express confusion around the difference between the D and the I. Relatedly, they struggle to identify what their specific D&I projects target to impact public health within the amorphous “black box” that is the singular, loosely defined “research-to-practice gap.” To improve conceptual clarity and enhance engagement with D&I science, we developed a graphic—the D&I Bridge—and an accompanying glossary of terms to use as a teaching and framing tool. The D&I Bridge depicts D&I science as bridging what we know from public health knowledge to what we do in public health practice with intention and equity, and it spans over four distinct, inter-related gaps: the public health supply gap, the public health demand gap, the methodological/scientific gap, and the expertise capacity gap. The public health supply gap is addressed by implementation strategies, whereas the public health demand gap is addressed by dissemination strategies. The methodological/scientific gap is addressed by producing generalizable knowledge about D&I, and the expertise capacity gap is addressed by developing the multi-disciplinary workforce needed to advance D&I. Initial practice feedback about the D&I Bridge has been positive, and this conceptualization of D&I science has helped inform our center’s D&I training, D&I project consultations, and strategic planning. We believe the D&I Bridge provides a useful heuristic for helping non-specialists understand the differential scopes of various D&I science projects as well as specific gaps that may be addressed by D&I methods.

Contributions to the literature

- Newcomers to dissemination and implementation (D&I) science often require guidance in two areas: (1) differentiating between the D and the I and (2) describing the specific public health issues addressed in their work.
- The D&I Bridge graphic and glossary define dissemination and implementation (D&I) science and differentiate the D from the I in lay language.
- The monolithic “implementation gap” is broken down into four inter-related gaps: public health supply, public health demand, methodological/scientific, and expertise capacity.
- The D&I Bridge is a useful tool for communicating about D&I science with non-specialists, facilitating D&I project consultations, and strategic planning around D&I priorities.

## Background

Interest in learning dissemination and implementation (D&I) science is at an all-time high [1]. Federal funding agencies are investing substantially in moving beyond a focus on discovery to a focus on late-stage translational science. In a program announcement soliciting D&I studies that spans 19 institutes (PAR-22–105), the National Institutes of Health (NIH) acknowledges that “closing the gap between biomedical or basic behavioral discovery, population health, and healthcare delivery and public health is both a complex challenge and an absolute necessity if we are to ensure that all populations benefit from the Nation’s investments in scientific discoveries.” When preparing this commentary, our team conducted a scan of the strategic plans of the 21 institutes across the NIH and found that 16 specified either “dissemination” or “implementation” as a key priority area; notably, 15 of these 16 participated in the program announcement calling for D&I proposals. Among those that did not *explicitly* state D&I science as a strategic priority, two institutes (National Human Genome Research Institute and National Institute on Allergy and Infectious Disease) emphasized the importance of integrating D&I into future strategic planning. This expansion of focus has been reflected in a plethora of funding announcements encouraging D&I proposals from the NIH and other federal funders, foundations, non-profits, and state departments of health. Not surprisingly, given this change in the funding climate, there has been a groundswell of interest from investigators eager to learn how to integrate D&I methods into their work [2, 3].

As the founding director and faculty/fellows of a Center for Dissemination and Implementation Science in a large integrated health system, we have experienced the surge of interest in D&I methods first-hand. Between the center’s first day on August 1, 2022, and our strategic launch meeting on November 15, 2022, we received over 100 requests for meetings and/or formal consultations relating to D&I. Our center program assistant tracked the first 100 requests for meetings and our center director and program assistant collaboratively classified the meeting objectives. Results indicated that 27% of the meetings/consults sought support on grant proposals, 27% sought strategic input as to how to infuse D&I methods into ongoing work s, 20% sought mentorship, 14% sought professional opportunities, and 12% sought training and education.

Over the course of our center’s initial (and continued) conversations, we have identified a key area of confusion among non-specialist investigators: the difference between the D and the I of D&I science. While a plethora of definitions of D&I science are readily provided by funders and in seminal manuscripts [4, 5], we have found that colleagues often have difficulty deciphering among and then applying the key components of these definitions. For instance, NIH PAR-22–105 defines dissemination research as “the scientific study of the targeted distribution of information and intervention materials to a specific public health, clinical practice, or policy audience” and implementation research as “the scientific study of the use of strategies to adopt and integrate evidence-based health interventions into clinical and community settings to improve individual outcomes and benefit population health.” Across our center’s first 100 meetings, our team fielded a set of recurring questions, including the following: how these definitions differed from activities in which colleagues were already engaging such as publishing, developing treatment manuals, and undertaking quality improvement initiatives; where activities such as training or education fit in these definitions; and whether specific strategies used in a study were addressing the D or the I. We discovered that for our consultations to be fruitful, non-specialists required a foundational understanding of how D and I differ.

A related area of confusion identified by our consultation team was identification and articulation of the specific challenge(s) that a given D&I project was trying to overcome. Often described in the field as an enormous “chasm” [6], the amorphousness of this characterization created challenges for non-specialists seeking to articulate the specific public health problems addressed in their project. Moreover, the vagueness of the singular “research-to-practice” gap often made it difficult for non-specialists to select the right D and/or I method(s) to guide their work.

To improve conceptual clarity and enhance the caliber of our D&I consultations, we have expanded the prevailing conceptualization of the singular research-to-practice gap to articulate four distinct, inter-related gaps that might be addressed by a single D&I project: the public health supply gap, the public health demand gap, the methodological/scientific gap, and the expertise capacity gap. Specification of distinct public health supply and public health demand gaps enables us to clearly differentiate between the D and the I. We have developed a graphic that introduces these four gaps along with a glossary defining each of the gaps in lay language.

Our primary goal in creating the graphic, called the D&I Bridge, was to provide a pragmatic resource for grant submissions, manuscripts, and D&I training materials to help non-specialists clearly and accurately communicate about the gap(s) they intend to address with their proposed research. A secondary benefit is that it has allowed us to organize our new center’s strategic planning and ensure that we intentionally engage in activities designed to address all four gaps in tandem.

## Applying the D&I Bridge

### Using the graphic and glossary

**Fig. 1:**
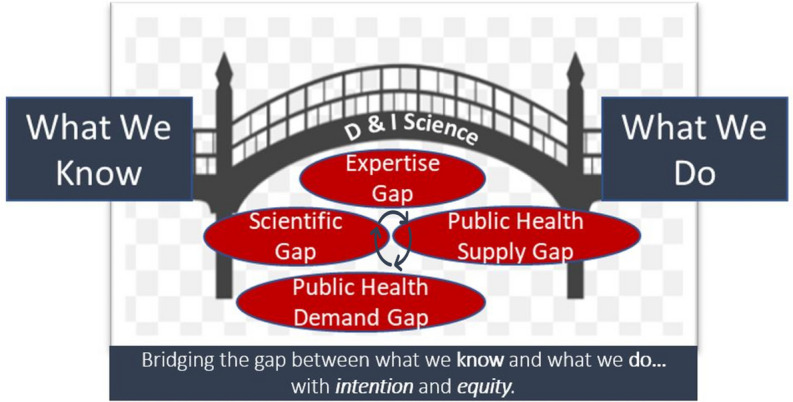
The D&I Bridge, a graphic depicting the gaps addressed by dissemination and implementation (D&I) science

The D&I Bridge (Fig. 1) represents D&I science as bridging the gap between public health knowledge, described simply as “What We Know,” and public health practice, described as “What We Do.” Under the bridge, we depict a banner that briefly defines D&I science as “bridging the gap between what we know (public health knowledge) and what we do (public health practice) with *intention* and *equity*.” Intention and equity are italicized to emphasize that implementation science initiatives must proactively and relentlessly center equity to avoid exacerbating existing disparities in clinical and community contexts. When we introduce this brief definition to non-specialists, we explain that while funding announcements and manuscripts often refer to the “research-to-practice” gap as a singular or monolithic thing, there are at least four inter-connected gaps that must be addressed, which are depicted in the graphic as circles.

The graphic is accompanied by a lay language glossary (Table 1). When using these teaching tools, we explicitly emphasize that while the graphic is two-dimensional and the definitions are presented in a specific order, we by no means intend to convey that the gaps are linear or causal. The overall bridge and each of the four gaps represent dynamic challenges for the field embedded within a complex reality that is constantly evolving. Moreover, the gaps are inter-connected, as indicated by the circle of arrows. Of note, we intentionally employ the familiar and highly simplistic economic concepts of supply and demand to facilitate understanding of complex concepts [7].

**Table 1:** Glossary of gaps addressed by dissemination and implementation (D&I) science

Specific gaps	Lay definition
Public health supply gap (addressed by the “I” in D&I Science)	• Gap between the *“care that could be”* if we used our best knowledge about what works and the *“care that actually is”* available in healthcare settings • Insufficient supply of effective health services in community and clinical settings
Public health demand gap (addressed by the “D” in D&I Science)	• Gap between *“those who need care”* and *“those who receive care”* • Insufficient demand for and access to effective health services by those who need such services
Methodological/scientific gap	• Gap between the methods needed to address the public health demand and public health supply gaps and the methods currently available
Expertise capacity gap	• Gap between the workforce needed to systematically address the public health and methodological/scientific gaps and the workforce currently available

The first gap we introduce is the public health supply gap. We point out that this is likely the gap with which our colleagues are most familiar. In lay language, we define it as the gap between the “care that could be” if we used our best public health knowledge about what works and the “care that actually is” available in healthcare settings: this definition is based on the description in the seminal Institute of Medicine report, “Crossing the Quality Chasm” [6]. Widely used synonyms are the “research-to-practice gap” or “evidence-to-practice gap” [8]. We explain that if we think about health services in familiar economic terms, this gap reflects the need to increase the *supply* of effective health services in clinical or community settings. To give an example of the consequences of this gap, we share the oft cited statistic (which our non-specialist colleagues are frequently alarmed by!) that it takes 17 years for 14% of research to improve the care of patients [9]. We then define *implementation science* as the identification of translatable methods to increase intentionally and equitably the public health supply. Such methods commonly include identification of barriers to the adoption of effective health services and the use of customized strategies to address these barriers [10, 11]. Our main point of emphasis is that the I in D&I science is designed to increase the supply of “care that could be” in public health contexts.

The second gap we present is the public health demand gap. This is a gap with which colleagues are typically less familiar, but we emphasize that it is equally important. In lay language, we describe it as the gap between “those who need care” and “those who receive care” [12]. Synonyms include “the treatment gap” or the “unmet need gap.” Again, using familiar economic terms, we explain that addressing this gap requires increasing the *demand* for effective health services. To give an example of the consequences of this gap, we share statistics noting the proportion of patients who meet diagnostic criteria for specific disorders who do not receive any care at all: for example, fewer than 20% of people with a substance use disorder and 50% of people with a mental health disorder will ever receive specialty care in their lifetime [13]. We then define *dissemination science* as the identification of translatable methods to increase intentionally and equitably the public health demand. Such methods commonly include identifying lack of knowledge or awareness of services among a specific population (or “consumer” as commonly described in the marketing literature [14]); understanding the target consumer’s preferences as to how they would like to receive information about effective health services [15, 16]; identifying structural and systemic barriers (including hierarchical structures and forms of oppression) that prevent consumers interested in seeking care from actually receiving such care [17]; and creating customized strategies to address both consumer-level preferences and structural-level barriers [18, 19]. Here, we emphasize that the D in D&I science is designed to promote demand for and reduce barriers to seeking services to equitably increase “those who receive care.”

The third gap we introduce is the methodological/scientific gap. We note that at the broadest level, this is the gap between the implementation science methods needed to quickly and efficiently address public health problems and the methods we currently have available. Addressing this gap requires knowledge generation and continuous methodological innovation in our implementation research [20]. D&I science is a young and developing field, and there is a need for methodological advances at multiple levels: we require innovation in how we apply and develop strategies to implement and disseminate new innovations, and we require innovation in how we design, conduct, and evaluate our research. The need for methodological innovation in our implementation research is perhaps not surprising when considering the historical lack of investment in this line of research. For example, a recent analysis of grants funded by the National Institute on Drug Abuse to address the opioid epidemic between 2015 and 2019 found that less than 2% of grants could be considered D&I research [21]; such low rates of funding reflect a need for greater investment in D&I science [22]. We assert that this gap is independent from the aforementioned gaps in public health supply and demand. For example, some D&I researchers may seek to address one of these public health gaps without an emphasis on methodological innovation (e.g., using well-established implementation strategies to implement an evidence-based intervention), whereas others may seek to focus on methodological innovation without an immediate focus on public health (e.g., developing new analytic strategies to improve the evaluation of multi-level, adaptive implementation strategies). In our admittedly biased view, the most exciting and impactful D&I work occurs at the intersection of one or more public health gaps and the methodological/scientific gap, via endeavors that aim to increase access to effective health services (public health demand or supply gap) while simultaneously promoting translatable methodological innovation that advances implementation research (methodological/scientific gap).

The fourth and final gap we define is the expertise capacity gap. We conceptualize this as the gap between the workforce needed to address the other three gaps—which includes both D&I scientists and D&I practitioners—and the workforce that is currently available. As a new center, we find that much of our efforts are designed to build the workforce of D&I scientists and practitioners, yet such efforts are not accounted for in traditional conceptualizations of the research-to-practice gap. Building the capacity of both the scientific and practice workforce is arguably a prerequisite to address and eventually close the other gaps of D&I. We believe that proactively addressing this gap is a highly significant area of contribution and that its explicit inclusion in our graphic serves to remind investigators of its strategic importance.

### Initial practice feedback

To date, we have leveraged these resources in a myriad of ways. Most notably, our center launched a formal D&I consultation service in Spring of 2023 and has used the D&I Bridge as a heuristic for organizing our consultations. Within the first 6 months of launching this new service, the D&I Bridge has provided scaffolding for consultations serving three distinct schools, four clinical affiliates, four medical divisions, and nine departments. Consultations have predominantly served investigators conducting research in the United States, with modest representation of global health researchers. Across our consultations, we have used the D&I Bridge (graphic and accompanying glossary) to establish a shared vocabulary between our consultants and consultees and to help colleagues who do not identify as implementation scientists to explicitly consider the potential public health impact of their work. As part of our attempts to foster continuous quality improvement, we have continually solicited feedback on the D&I Bridge with an eye towards making refinements. Feedback from global health researchers suggests that the graphic and glossary resonate and that the gaps are viewed as even more pronounced in low- and middle-income countries. Feedback from researchers across settings directly led to the creation of the banner under the bridge that emphasizes the need to center intention and equity.

**Fig. 2:**
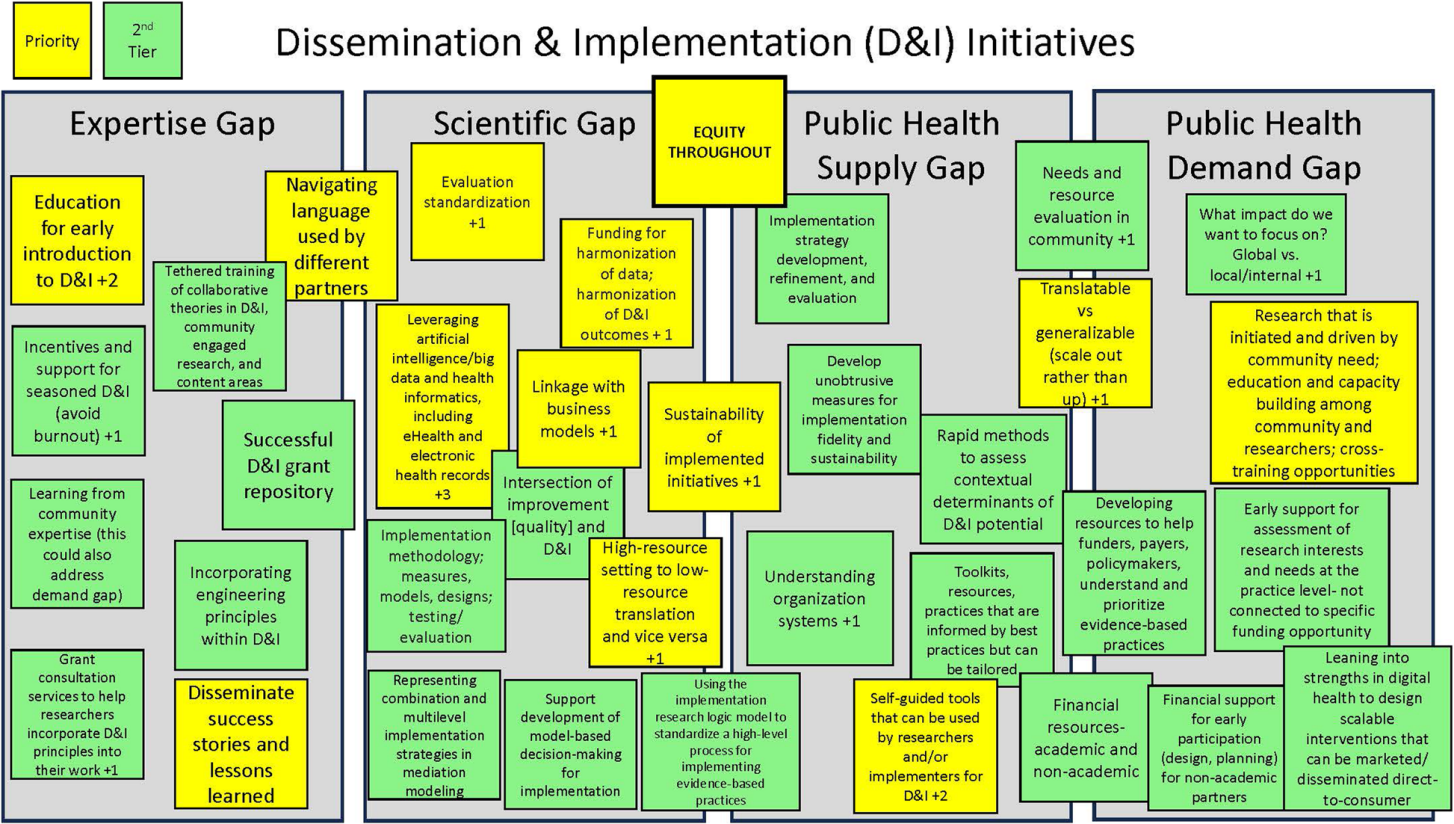
Results of an interactive exercise mapping strategic priorities for our center onto each of the four gaps

We have also used the D&I Bridge throughout our center’s strategic planning process. For instance, we used an initial draft of the graphic in the strategic launch event with over 100 institutional leaders (e.g., medical school department chairs, center/institute directors throughout our health system) as well as in a series of workgroup meetings focused on identifying D&I priorities across our institution. Utilization of the D&I Bridge has had two key benefits. First, it has helped to center colleagues around the different gaps that our center and the field of D&I science seek to address. Second, it has enabled us to map our partners’ strategic feedback onto each of the specific gaps to ensure our center’s actions maintain a focus on enhancing public health impact. As an example, during one of our center’s workgroup meetings, we completed an interactive, virtual Jamboard exercise to encourage the workgroup members to map their strategic priorities for our center onto each of the four gaps; a snapshot of the actual results of this strategic exercise is presented in Fig. 2.

Finally, we have routinely used the D&I Bridge in presentations both within and outside of our institution, to provide user-friendly conceptual grounding for audience members. In our center’s first year of operation, we used the D&I Bridge in 26 invited national and regional talks. Some feedback on these presentations have included “this is the most clear overview of D&I science I have seen in all of my years in the field,” “incredibly clear and well-put together,” and “tremendously clear introduction!”.

## Conclusions

Our hope is that the D&I Bridge and accompanying glossary will be useful for D&I enthusiasts and non-specialists seeking to clearly articulate the difference between the D and the I and to understand how these areas of science map on to specific gaps addressed in their research. We believe that the ability to succinctly articulate the public health problems addressed in a specific study is a vital precursor to communicating the benefits of the work via comprehensive frameworks such as the Translational Science Benefits Model, which identifies 30 potential benefits of clinical and translational research for four different audiences [23]. We encourage colleagues to freely use the D&I Bridge in their teaching and scholarship, in whatever manner is most useful. Most importantly, we encourage colleagues to use the D&I Bridge by making additions, substitutions, or modifications to fit their needs. For instance, after the strategic Jamboard exercise described above, our workgroup made the decision to adapt the graphic by adding three layers of water to better illustrate three of our institutional values of equity, translational science, and pragmatism. This modification enabled us to demonstrate three core principles that underlie our institution’s efforts to bridge the gap between public health knowledge and practice.

We recognize that the D&I Bridge is of a limited scope—the graphic and glossary address the narrow issue of *what* issues D&I investigators commonly seek to address without providing any guidance as to the *how*. Our view is that having conceptual clarity about D&I science is necessary, but not sufficient, to optimize public health impact. Future directions of this work include expansion of the D&I Bridge to offer a set of case examples of initiatives addressing each of the gaps with specific implementation strategies.

In a seminal 2020 article, Geoff Curran shared a simple teaching tool to help investigators understand the difference between an intervention and implementation strategy [4]. He concluded his manuscript by asserting: “given the complexity of implementation science, providing a clear definition of it…can be difficult” [4]. We could not agree more with this assertion. By freely disseminating simple, pragmatic resources, we hope to stimulate discourse on how to best address the vexing problem of defining the D and the I to engage non-specialists in the field of public health. We also aspire to help D&I enthusiasts to precisely articulate the gaps addressed in their work as an essential step towards increasing public understanding of and support for D&I science.

## Data Availability

The only data reported are the qualitative description of consultations provided by the Center for Dissemination and Implementation Science. These data are available from the lead author by reasonable request.

## Abbreviation

D&I: Dissemination and implementation
